# Neural activity of the auditory cortex predicts speech recognition of patients with asymmetric hearing loss after cochlear implantation

**DOI:** 10.1038/s41598-022-12139-y

**Published:** 2022-05-16

**Authors:** Iva Speck, Susan Arndt, Johannes Thurow, Alexander Rau, Antje Aschendorff, Philipp T. Meyer, Lars Frings, Ganna Blazhenets

**Affiliations:** 1 Department of Otorhinolaryngology - Head and Neck Surgery, Medical Center, Faculty of Medicine, University of Freiburg, Killianstraße 5, 79106 Freiburg, Germany; 2 Department of Nuclear Medicine, Medical Center, Faculty of Medicine, University of Freiburg, Hugstetter Straße 55, 79106 Freiburg, Germany; 3 Department of Neuroradiology, Medical Center, Faculty of Medicine, University of Freiburg, Breisacher Str. 64, 79106 Freiburg, Germany; 4 Department of Diagnostic and Interventional Radiology, Medical Center, Faculty of Medicine, University of Freiburg, Hugstetter Straße 55, 79106 Freiburg, Germany

**Keywords:** Auditory system, Neural circuits

## Abstract

Patients with asymmetric hearing loss show an asymmetry of glucose metabolism of the primary auditory cortex (PAC). We investigated whether this asymmetry could serve as an objective predictor for speech recognition with CI. Nine patients underwent ^18^FDG PET prior to CI surgery. Average normalized ^18^FDG uptake of 25% of voxels with highest uptake was calculated for the PAC employing a probabilistic atlas and cerebellar cortex as reference. Differences in glucose metabolism of the PAC were assessed by an asymmetry index (AI-PAC). We tested the correlation between outcome of CI surgery (6 months post implantation), AI-PAC and clinical predictors. Pre-operative AI-PAC showed a positive correlation with speech recognition with CI (significant for sentences and numbers; trend for monosyllabic words). With a pre-operative AI-PAC ≥ 4.2%, patients reached good CI outcome in sentence recognition of 59–90% and number recognition of 90–100% and less favorable CI outcome in monosyllabic word recognition of 25–45%. Age at symptom onset was significantly associated with all measures of speech recognition, while deafness duration was only associated with sentence recognition. AI-PAC allows for a reliable and quantitative pre-operative prediction of early improvement in speech recognition after CI. ^18^FDG PET may be a valuable addition to the objective pre-operative assessment of CI candidates. Further studies in larger cohorts and with longer follow-up times are needed.

## Introduction

Asymmetric hearing loss (AHL) and its extreme form single-sided deafness (SSD) impair speech understanding in background noise^1–4^, localization of sound sources^1,2,4,5^, and cause exhaustion, frustration, and social isolation as a result of increased stress levels and increased listening effort^4,6–8^. A cochlear implant (CI) is the only therapy that can improve binaural hearing and therefore speech recognition in background noise and localization of sound sources in subjects with AHL and SSD^1,9–12^. Patients to receive CI are evaluated by a pre-operative workup consisting of: (1) anamnesis and ENT medical examination, (2) subjective hearing tests (e.g. pure tone and speech audiometry in quiet and noise), (3) objective hearing tests (e.g. brainstem evoked response audiometry), (4) evaluation of hearing-related quality of life, (5) balance tests and (7) imaging techniques (CT and/or MRI)^13–15^. Predictions of CI outcome are mostly based on the context of all data collected during pre-operative workup and the duration of deafness^16^. However, the results of CI recipients with AHL and SSD differ considerably between subjects^16–19^. Given the invasiveness and high costs of CI, there is a high need for a valid predictor of CI outcome in subjects with AHL. Although various clinical and audiological parameters are widely available, they have proven to be of only limited value^16^.

In a prior study, we showed that AHL and SSD alter glucose metabolism [as a measure of neuronal activity] of the contralateral colliculus inferior and primary auditory cortex (PAC)^20^. In addition, the degree of asymmetry of glucose metabolism between ipsi- and contralateral PAC varied between subjects. With longer duration of deafness, the initially reduced activity in the auditory cortex contralateral to the more hearing-impaired ear increased^20^, so that asymmetry of PAC decreased with time. Thus, we hypothesized that these changes might be due to cross-modal takeover of the PAC by other sensory systems (e.g., visual or vibrotactile system)^21,22^. Therefore, the outcome with CI can possibly be predicted before actual implantation. The aim of this study was to examine whether the neuronal activity in the PAC assessed by ^18^FDG PET predicts speech recognition with CI.

## Methods and materials

### Subjects

Approval by the local ethics committee of the Freiburg University (vote 330/18, DRKS00015477) was granted and all subjects gave written informed consent. The present study was conducted in accordance with the guidelines of the Declaration of Helsinki (Washington, World Medical Association, 2013). We retrospectively included nine right-handed CI recipients with AHL or SSD (mean age 46 ± 20 years) who received a pre-surgical ^18^FDG PET for clinical indication (mostly, exclusion of other neurological conditions). Characteristics of included CI recipients are given in Table 1. Four patients with AHL wore a hearing aid on the better-hearing ear. No hearing aids were worn in the poorer-hearing ear. All subjects reported normal vision.

**Table 1:** Results of the audiological tests along with the demographic characteristics of the included patients.

ID	Age at CI operation (years)	Sex	Hearing impaired ear	Etiology of asymmetric hearing loss	Duration of deafness (years)	Pre- vs. postlingual SSD/AHL	Bone conduction PTA4 (dB HL)		Air conduction PTA4 (dB HL)		Monosyllabic word recognition at 65 dB SPL (%)		Speech recognition with CI at 65 dB SPL (%) in quiet 6 months after initial fitting		
							Better-hearing ear	Poorer-hearing ear	Better-hearing ear	Poorer-hearing ear	Better-hearing ear	Poorer-hearing ear	OLSA	Monosyllabic word recognition	Number recognition
1^b^	36.3	♂	R	Sudden hearing loss	1.5	Postlingual	3	95	6	> 130	100	0	90	35	100
2^b^	52.8	♂	L	Intra-cochlear schwannoma	12	Postlingual	15	> 130	15	> 130	100	0	58	35	80
3^b^	76.7	♂	R	Sudden hearing loss	0.7	Postlingual	20	> 130	39	> 130	95^a^	0	59	30	90
4^b^	60.6	♂	R	Sudden hearing loss	21	Postlingual	41	114	44	114	70^a^	0	75	45	100
5^b^	18.2	♀	L	Large aquaeductus vestibuli syndrome	15	Perilingual	13	> 130	18	> 130	100	0	7	0	10
6	54.8	♂	R	Unknown	49	Perilingual	56	> 130	59	> 130	55^a^	0	0	5	40
7	28.5	♀	R	Large aquaeductus vestibuli syndrome	28	Perilingual	21	74	24	86	90	0	4	5	10
8	19.2	♂	L	Sudden hearing loss	1	Postlingual	3	> 130	7	> 130	100	0	59	25	90
9	56.8	♂	R	Fracture of right petrous bone	28	Postlingual	37.5	> 130	42.5	> 130	75^a^	0	47	35	100

^a^Hearing aids used on the better-hearing ear.

^b^Subjects reported in Speck et al.^20^.

### Hearing tests

We included hearing tests performed six months after initial fitting of the CI. Audiometry tests of speech recognition at 65 dB SPL (decibel sound pressure level) included monosyllabic word recognition in the Freiburg intelligibility test^23^, number recognition in the Freiburg intelligibility test^23^ and the Oldenburg sentence test (OLSA)^24,25^ in quiet. All audiometry tests were performed in a sound-proof booth using the Auritec AT 1000 device. Speech material was presented via headphones to the better-hearing ear and via audio cable to the CI.

The Freiburg intelligibility test^23^ consists of 10 groups of 20 monosyllabic words each and 10 groups of 10 two-digit numbers each. We presented one group of words and one group of numbers at 65 dB SPL. The participants were asked to repeat the words or numbers and guess if unsure. Adults with normal hearing are expected to repeat 100% words and numbers correctly at 65 dB SPL^26^.

The 5-word-sentences of the OLSA are randomly combined from ten possible words: name—verb—numeral—adjective—object resulting in sentences that are grammatically correct but semantically unpredictable. The participants were asked to repeat the sentences and guess words if unsure. Adults with normal hearing are expected to repeat 100% of sentences correctly at 65 dB SPL^26^. Higher scores (in % correct) in all three tests indicate better performance.

### ^18^FDG PET

All PET scans were performed on a fully digital high-resolution clinical PET/CT system (Philips Vereos, Philips Healthcare, The Netherlands). PET acquisition and processing was performed as previously described^20^. In brief: Prior to PET, patients fasted for at least 6 h and were intravenously injected with 207 ± 24 MBq ^18^FDG (euglycaemia). At 50 min after injection under resting conditions (eyes open, ambient noise), a static 10-min PET scan was acquired. During the first 50 min after injection 4 patients with AHL wore a hearing aid on their better ear to mirror their every-day hearing situation (i.e., highest asymmetry between auditory inputs; see Table 1). Scans were spatially normalized to the Montreal Neurological Institute (MNI) space using SPM12 (https://www.fil.ion.ucl.ac.uk/) and an in-house ^18^FDG PET template. In analogy to Speck et al.^20^, averaged ^18^FDG uptake of the 25% of voxels with the highest uptake within the PAC was calculated after proportional scaling of individual PET data to the uptake in cerebellar cortex. The PAC volume of interest (VOI) was taken from Morosan et al.^27^ and comprised the subregions Te1.0, Te1.1, and Te1.2 (50% probability for PAC, smoothed with 4 mm and again thresholded at 50% probability to reach scanner resolution). The cerebellar cortex VOI was taken from the SUIT atlas^28^.

Individual differences in normalized ^18^FDG uptake of the PAC were assessed by an asymmetry index (AI-PAC; ipsi- minus contralateral side [referring to the more hearing-impaired ear] divided by their mean). The reference value of AI-PAC was derived from the control population as the upper limit of the 95% confidence interval of the absolute value of AI-PAC. As a control sample, ^18^FDG PET scans of healthy individuals (recruited by local advertisement; no neurologic or psychiatric condition, unimpaired neuropsychological evaluation, normal MRI of the brain) with normal hearing acquired under identical conditions with the same PET system were evaluated (n = 13; mean age 68 ± 7 years, mean AI-PAC [95% CI] = 2.9 [1.7–4.2]). Of note, given the recent advent of fully-digital PET/CT system, the currently available control sample showed higher mean age than the patient sample (68 vs. 46 years, for patients and controls, respectively; p < 0.01). However, a confirmatory analysis of an age-matched cohort acquired with a different state-of-the-art, conventional PET/CT scanner showed that the AI-PAC is stable across age (e.g., n = 8 healthy controls (sub-cohort from Baumgartner et al. ^29^), 44 ± 9 years, AI-PAC [95% CI] = 3.22 [1.7–4.7]). The AI-PAC was not significantly different between control cohorts (p > 0.1) and there was no significant association with age, neither within nor across cohorts (all p > 0.1).

### Magnetic resonance imaging

All patients received cerebral MRI with special focus on the structures of the inner ear and the auditory pathway. The delay from MRI to PET examinations was on average 78 ± 76 days. All MRI scans were re-evaluated by an experienced radiologist (5 years of experience in neuroimaging). Special attention was paid to the PAC^27^, the course of the auditory pathway^30^, and the inferior colliculi.

### Non-imaging predictors of CI outcome

Deafness duration and age at symptom onset were evaluated as commonly used predictors of CI outcome. The onset of deafness was defined as the time at which a hearing aid was no longer worn on the poorer-hearing ear because of the lack of benefit. Deafness duration and age at symptom onset were primarily treated as continuous variables. For exploratory reasons, deafness duration was split to short and long duration based on the median of the cohort. Age at symptom onset was additionally dichotomized into ‘perilingual’ vs. ‘postlingual’ SSD and AHL (see Table 1).

### Statistics

Pearson correlation analyses were performed to estimate the strength of relationship between each of the hearing tests (OLSA, monosyllabic word recognition and number recognition) and non-audiological parameters (1: AI-PAC; 2: duration of deafness; and 3: age at symptom onset) as well as among non-audiological parameters. As for clinical decision making it is common to use binary stratifiers, we calculated effect size (Cohen’s d) between binary groups for each predictor. Statistical analyses were performed using R software (https://www.R-project.org/).

## Results

### ^18^FDG PET

**Figure 1:**
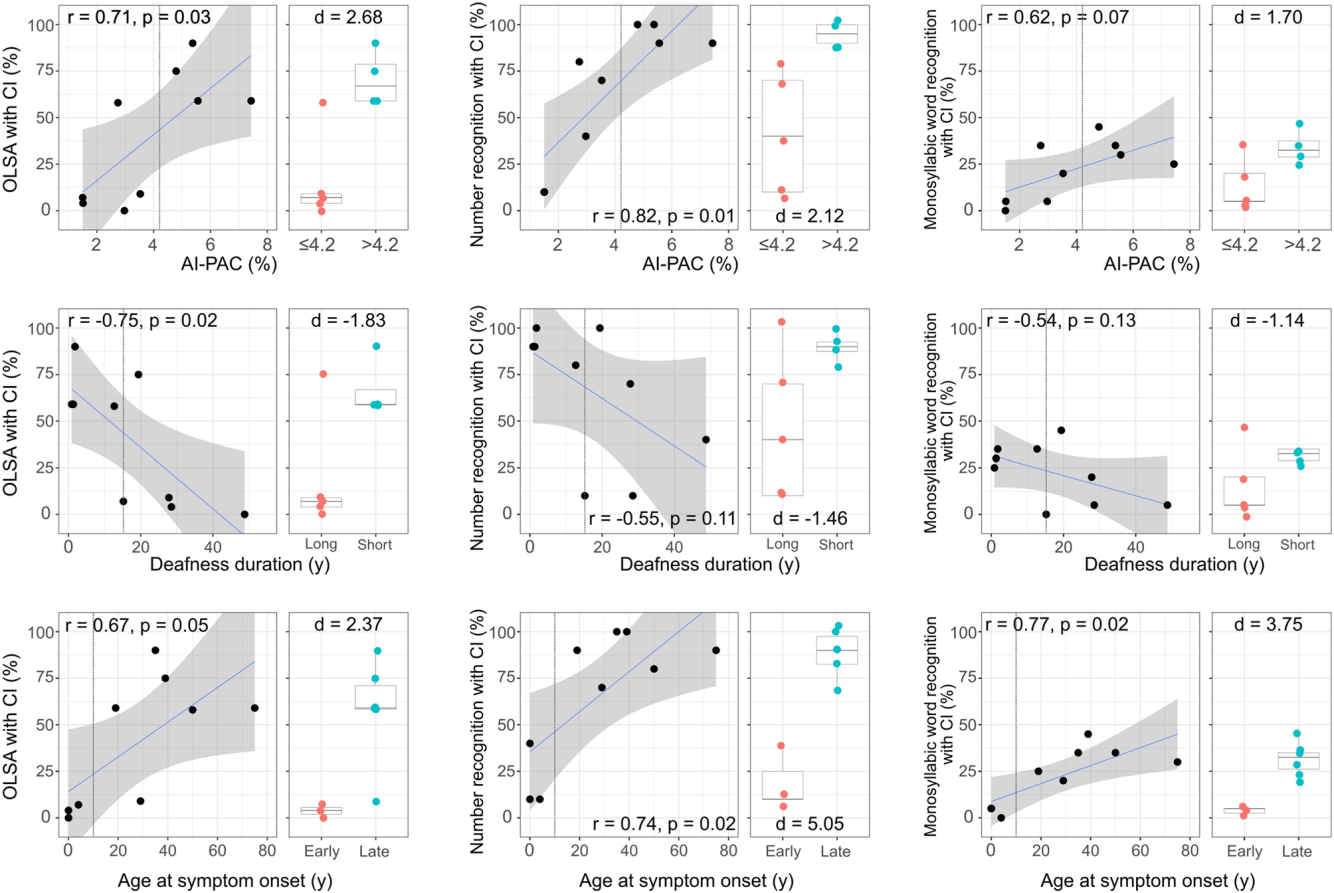
Associations between asymmetry index (AI) of the primary auditory cortex (PAC), deafness duration and age at symptom onset with the hearing tests: sentence recognition (OLSA), number recognition and monosyllabic word recognition with cochlear implant (CI) six-month post implantation. Each dot represents individual data. Dashed line shows corresponding cutoffs. Linear regression analysis displayed as regression line (blue) and 95% confidence interval (grey area). *r* Pearson correlation coefficient, *d* Cohen’s d.

Pre-operative AI-PAC showed a significant positive correlation with sentence recognition (OLSA; r = 0.71, p = 0.03, t = 2.68) and number recognition (r = 0.82, p = 0.006, t = 3.83), and trend-level association to monosyllabic word recognition (r = 0.62, p = 0.07, t = 2.11) with CI six months after initial fitting. For patients with AI-PAC ≥ 4.2%, OLSA and numerical recognition with CI were 59–90% and 90–100%, respectively, indicating good to excellent CI outcome (Fig. 1, upper panel). For patients with AI-PAC ≥ 4.2%, monosyllabic word recognition was 25–45%, indicating less favorable CI outcome. There was only trend-level association of AI-PAC to deafness duration (r = −0.61, p = 0.08) and no significant association between AI-PAC and age at symptom onset (p > 0.1). In contrast to AI-PAC, we found no association between mean normalized ^18^FDG uptake on the contralateral (referring to the more hearing-impaired ear) PAC and audiological tests, disease duration or age at symptom onset (all p > 0.1).

For clinical decision making the use of binary stratifiers is prevalent. Group split by AI-PAC (above vs. below the upper limit of the normal range, i.e. AI-PAC ≥ 4.2% vs. < 4.2%) showed very large group difference in OLSA (mean of 70.75% vs. 15.60%, d = 2.68), number recognition (95% vs. 45%, d = 2.12) and monosyllabic word recognition (33.75% vs. 13.00%, d = 1.70), respectively (Fig. 1, upper panel).

### Magnetic resonance imaging

No relevant asymmetry, signal alteration, or atrophy was found in any patient. Beyond that, no other structural changes (e.g., vascular, gliotic or inflammatory) were observed. Visual and voxel-wise assessments of healthy controls revealed no pathological findings.

### Deafness duration

Pre-operative deafness duration was significantly negatively associated with OLSA with CI (r = −0.75, p = 0.02, t = −2.99). Neither number recognition nor monosyllabic word recognition were associated with deafness duration (all p > 0.1, see Fig. 1, middle panel) and only a trend-level association between deafness duration and age at symptom onset was observed in this cohort (r = −0.59, p = 0.08).

In addition, we used a median split of deafness duration as a binary predictor as done in clinical routine. Groups stratified by deafness duration (above vs. below median, i.e. duration ≥ 15.20 vs. < 15.20 years) showed large to very large differences in OLSA (mean of 19.00% vs. 66.50%, d = −1.83), number recognition (46.00% vs. 90.00%, d = −1.46) and monosyllabic word recognition (15.00% vs. 31.25%, d = −1.14), respectively (Fig. 1, middle panel).

### Age at symptom onset

Age at symptom onset was significantly positively associated with OLSA (r = 0.67, p = 0.046, t = 2.41), number recognition (r = 0.74, p = 0.02, t = 2.95) and monosyllabic word recognition (r = 0.77, p = 0.015, t = 3.18), see Fig. 1 (bottom panel).

Based on perilingual vs. postlingual SSD and AHL (a binary predictor commonly used in clinical routine), groups showed very large differences in OLSA performance (58.33% vs. 3.66%, d = 2.37), number recognition (88.33% vs. 20.00%, d = 5.05) and monosyllabic word recognition (31.66% vs. 3.33%, d = 3.75), for onset perilingual vs. postlinugal SSD and AHL, respectively.

## Discussion

In the present study, the asymmetry of neuronal activity of the auditory cortex as assessed by ^18^FDG PET was examined as a possible objective predictor of CI outcome in patients with AHL and SSD. The AI-PAC before CI was associated with performance in the audiological tests six month after initial fitting of CI indicating that better early outcome was associated with higher AI-PAC, i.e., greater neuronal activity of the ipsi- relative to the contralateral PAC. Moreover, for patients with AI-PAC exceeding the asymmetry that was present in a control cohort, 6-month follow-up examinations showed good to excellent performance in sentence and number recognition here, emphasizing the value of the ^18^FDG PET as a predictor of early CI outcome in patients with AHL. These patients, with a pre-operative AI-PAC ≥ 4.2%, showed better monosyllabic word recognition than those with a lower AI-PAC. Nevertheless, monosyllabic word recognition was poorer than expected for 6-months follow-up examination in both groups^31–33^.

Previously, our group has shown that asymmetric hearing loss has a significant impact on glucose metabolism of the auditory pathway^20^. Moreover, hypometabolism in the auditory cortex contralateral to the more hearing-impaired ear diminished in patients with longer duration of hearing impairment^20^, possibly indicating a reorganization at the cortical level. Similar effects were shown by Han et al.^34^ in 37 patients with bilateral deafness: the hypometabolism in both auditory cortices decreased with longer duration of deafness. Driven by these findings, we hypothesized that the asymmetry of the glucose metabolism will inversely reflect the level of cortical reorganization in the PAC.

Our results and conclusions are strengthened by the literature on subjects with bilateral deafness. Lee et al.^35^ showed a predictive value of the glucose metabolism in prefrontal cortex and superior temporal gyrus on CI outcome in 33 children with congenital bilateral deafness using ^18^FDG PET. In 2021, Sun et al.^36^ used preoperative MRI to demonstrate that outcome with CI in subjects with bilateral hearing impairment can be predicted by gray matter volume in the left superior, middle temporal cortex and bilateral thalamus. In contrast, Han et al.^34^ found that the metabolism of the PAC did not correlate with CI outcome in subjects with bilateral deafness and interpreted the lack of prognostic value as a sign of resistance of the PAC to cross-modal plasticity in patients with postlingual bilateral deafness. In the cohort of patients with AHL and SSD presented in the current study, the metabolism of the PAC contralateral to the more hearing-impaired ear similarly to Han et al.^34^ did not correlate with CI outcome, while the AI-PAC indeed showed good prognostic value.

Duration of deafness in AHL and SSD is ill-defined and in general is a highly subjective measure that relies heavily on individual disease burden and patient’s memory. Nevertheless, it is widely used and has been proven to be a predictive factor of CI outcome in subjects with bilateral deafness^37–40^. In most studies in subjects with bilateral deafness, the age of deafness onset is set to the time when oral communication becomes impossible even with well-fitted hearing aids. This is, however, not feasible for patients with AHL and SSD, as an oral communication is further possible using the better-hearing ear. The onset of deafness is therefore defined as the time at which a hearing aid is no longer worn on the poorer-hearing ear because of the lack of benefit. Ambiguity in determining the time of the disease onset and the respective deafness duration can among others explain limited association to the post-operative outcome. This underlines the possible advantage of the AI-PAC as an objective predictor that is not dependent on the memory or cooperation of the patient.

Similarly, to deafness duration, age at symptom onset is not usually a reliable measure. Especially difficult is the definition of age at symptom onset in childhood for SSD and AHL. Before the obligatory bilateral newborn hearing screening (in Germany since 2009), children with prelingual SSD were usually not diagnosed before they reached school age^41,42^. In this study, the reported age at symptom onset for patients with SSD/AHL onset in childhood heavily relies on informal reports of their parents. Nevertheless, we found a positive correlation between age at deafness onset and speech recognition with CI: patients with later disease onset showed stronger benefit from an implantation. As discussed by Han et al.^34^, deafness at an older age may lead to less cross-modal plasticity of the auditory cortex. This hypothesis is further promoted by findings of Lee et al.^35^ in subjects with prelingual bilateral deafness. In support of this theory, two participants with perilingual AHL included in the present study had low AI-PAC. These findings further support a stronger plasticity at younger age. A prompt therapy with CI may therefore be advisable in young patients with AHL or SSD.

Besides deafness duration and age at symptom onset, various alternative markers have been proposed as predictors of CI outcome in patients with bilateral deafness: age^38^, degree of residual hearing^38^, tinnitus^43^, surgical technique^44^ and pre-existing vestibulopathy^45^. In contrast, the literature of predictive factors in subjects with SSD and AHL is sparse. A meta-analysis by Kitterick and Lucas^16^ (although in a limited sample of only 34 adults with SSD) showed a significant negative association between speech recognition in noise with CI and duration of deafness and a significant positive correlation with residual hearing in the implanted ear^16^. As only two included subjects showed residual hearing in the poorer-hearing ear in the present study, we were not able to examine the impact of residual hearing on CI outcome in the present study.

Patients with longer durations of deafness are usually considered as borderline candidates for CI. Nevertheless, some perilingual patients with long disease duration reported in this study have been considered for CI implantation based on the following considerations: First, the earliest reported date has been chosen as time of symptom onset and, therefore, the duration of hearing impairment is likely to be overestimated. Second, functional integrity of the auditory nerves in these patients was confirmed by promontory tests. Promontory test was performed by placing an electrode through the local anesthetized eardrum on the promontory to electrically stimulate the auditory nerve. A hearing impression during stimulation, gap detection during hearing impression^46,47^ and the temporal difference limen^39,47^ was assessed to estimate functional intactness of the auditory nerve. Only those perilingual onset patients in whom promontory test indicated functional intactness of the auditory nerve were considered as candidates for CI. Despite the possible limited outcome in patients with longer hearing impairment, possible (SSD) or starting hearing impairment (AHL) of the better-hearing ear was one of the main reasons to decide for an implantation in all included patients. The negative effect of SSD on the hearing capacity of the better-hearing ear as well as a possible protective effect of a CI were previously reported by Arndt et al.^48^. Nevertheless, perilingually deafened patients are still borderline candidates for a CI and are therefore extensively consulted on a possibly limited outcome of implantation. Additional predictive factors like the one investigated in the present study cortex could further improve the indication and examination in such borderline candidates.

We decided to report hearing measurements six months after initial fitting of the speech processor of the CI to test our hypothesis that pre-operative AI-PAC is correlated to outcome with CI. Still, six months of CI experience is a relatively short time to assess final outcome.

Cusumano et al.^49^ showed that subjects with pre- and postlingual deafness have a performance peak six months after initial fitting of their CI. Nevertheless, ongoing improvements are still reported twelve months after initial fitting^11,49–51^ and in some tasks (e.g. subjective spatial hearing) even later^10,12^. Thus, the present relatively short follow-up time limits the predictive value of the AI-PAC to *early* outcome with CI. In particular, this applies to patients with early onset, in whom improvements with CI may require more time^49^. We are therefore planning to further validate our results in one, two, and five years after initial fitting.

This study is further limited in number of enrolled patients. Given the heterogeneity of the cohort in terms of etiology, disease onset and duration, the results need to be reproduced in larger studies. Moreover, the potential value of proposed predictor has to be separately addressed in pre- and postlingual patients.

Spatial hearing tests and OLSA in noise were not available for all included subjects six months after initial fitting of the CI. Therefore, we did not include these audiometric tests in our study. In future, prospective studies including a higher number of participants’ spatial hearing tests and speech recognition in background noise are planned at 6 months after initial fitting and yearly afterwards.

In conclusion, pre-operative asymmetry of the glucose metabolism in the PAC assessed by ^18^FDG PET may serve as a potential predictor of early post-operative CI outcome and employed as unbiased objective precision medicine tool in patients with AHL and SSD. Studies with a larger number of subjects and longer follow-up time are necessary to confirm our hypothesis.

## Data Availability

The data generated and analyzed during the current study are not publicly available but could possibly be provided by the corresponding author on reasonable request and upon approval of the local ethics committee.
